# Neutrophil to Lymphocyte Ratio as a Predictor of Postoperative Outcomes in Traumatic Brain Injury: A Systematic Review and Meta-Analysis

**DOI:** 10.3390/diseases11010051

**Published:** 2023-03-15

**Authors:** Andrew Nguyen, Alexander Nguyen, Timothy I. Hsu, Harrison D. Lew, Nithin Gupta, Brandon Nguyen, Akhil Mandavalli, Michael J. Diaz, Brandon Lucke-Wold

**Affiliations:** 1 College of Medicine, University of Florida, Gainesville, FL 32601, USA; 2 School of Medicine, University of California, Irvine, CA 92617, USA; 3 School of Medicine, Campbell University, Lillington, NC 27546, USA; 4 Alix School of Medicine, Mayo Clinic, Scottsdale, AZ 85054, USA; 5 Department of Neurosurgery, University of Florida, Gainesville, FL 32608, USA

**Keywords:** neutrophil to lymphocyte ratio, traumatic brain injury, neurological deficit, adverse outcomes, neurosurgery

## Abstract

(1) Introduction: Traumatic brain injury (TBI) is a leading cause of injury and mortality worldwide, carrying an estimated cost of $38 billion in the United States alone. Neutrophil to lymphocyte ratio (NLR) has been investigated as a standardized biomarker that can be used to predict outcomes of TBI. The aim of this review was to determine the prognostic utility of NLR among patients admitted for TBI. (2) Methods: A literature search was conducted in PubMed, Scopus, and Web of Science in November 2022 to retrieve articles regarding the use of neutrophil to lymphocyte ratio (NLR) as a prognostic measure in traumatic brain injury (TBI) patients. Inclusion criteria included studies reporting outcomes of TBI patients with associated NLR values. Exclusion criteria were studies reporting only non-primary data, those insufficiently disaggregated to extract NLR data, and non-English or cadaveric studies. The Newcastle-Ottawa Scale was utilized to assess for the presence of bias in included studies. (3) Results: Following the final study selection 19 articles were included for quantitative and qualitative analysis. The average age was 46.25 years. Of the 7750 patients, 73% were male. Average GCS at presentation was 10.51. There was no significant difference in the NLR between surgical vs. non-surgical cohorts (SMD 2.41 95% CI −1.82 to 6.63, *p* = 0.264). There was no significant difference in the NLR between bleeding vs. non-bleeding cohorts (SMD 4.84 95% CI −0.26 to 9.93, *p* = 0.0627). There was a significant increase in the NLR between favorable vs. non-favorable cohorts (SMD 1.31 95% CI 0.33 to 2.29, *p* = 0.0090). (4) Conclusions: Our study found that NLR was only significantly predictive for adverse outcomes in TBI patients and not surgical treatment or intracranial hemorrhage, making it nonetheless an affordable alternative for physicians to assess patient prognosis.

## 1. Introduction

Traumatic brain injury (TBI) is a common cause of brain damage with both mild and severe initial presentations contributing to long-term sequelae in all ages. In its mildest form, TBI may present with headache and in severe forms can cause comatose states and death [1]. Additional features of TBI that may be present include nausea/vomiting, tinnitus, loss of consciousness (LOC), neurological deficits, agitation, amnesia, pupillary changes, hypotension, and seizure [1]. In the United States, TBI represents approximately 40% of deaths due to acute injury and is the leading cause of death in individuals younger than 45 years old [2]. In addition, TBI also carries an enormous financial burden with an estimated total annual cost of $37.6 billion, $12.7 billion of this accounting for lost income due to premature death [3].

Blunt trauma and penetrating mechanisms can cause TBI, however, both contribute to tissue damage, alteration in cerebral blood flow, vasospasm, and release of inflammatory mediators—ultimately leading to edema and cell death [4]. While the primary insult to the brain is a cause of major concern, prevention of secondary damage is a major concern as well. Initially, patients may be evaluated using the Glasgow Coma Scale (GCS) in order to determine the extent of injury and risk factors for deterioration [5]. This widely used grading scale is often used in conjunction with imaging studies to evaluate a patient for neurosurgical intervention. Due to the secondary effects of TBI such as intracranial bleeding and increased intracranial pressure, neurosurgical intervention may be required to evacuate hematomas and/or relieve swelling with decompressive craniotomy [6]. Although the GCS score has been established as a relatively accurate prognostic indicator, it is a subjective measure that has been shown to have inter-observer variability [7,8]. Furthermore, GCS does not provide information regarding inflammatory processes within the brain that may cause acute deterioration in patients, thus there is a need to investigate a more objective prognostic measure of TBI patients.

Recently, the use of the neutrophil to lymphocyte ratio (NLR) has been investigated as a reliable marker for response to organ dysfunction, disease, and tissue injury [9]. With multiple reports using NLR as an accurate indicator for outcomes in diseases such as COVID-19, cancers, and stroke, there have also been early reports using NLR in the setting of TBI [10,11,12,13,14,15,16,17]. During TBI, neutrophils are recruited to brain injury and play a role in releasing inflammatory cytokines, free radicals, and proteases which play a role in the pathogenesis of secondary damage [18]. Unlike neutrophils which are among the first to respond to TBI, the role of lymphocytes is not as clear. Current data suggests that lymphocytes do not respond to TBI within the first week of injury [19]. Furthermore, unlike neutrophils which increase tissue damage, T lymphocytes may induce a healing process in the damaged brain [20]. These differing temporal responses and functions indicate that a higher NLR (higher neutrophils and lower lymphocytes) may predict worse outcomes in TBI patients. Currently, studies regarding NLR in TBI have demonstrated that higher NLR is an independent prognostic factor for mortality in severe TBI [17,20,21]. However, due to the relatively new implementation of this test, there remains no large-scale statistical analysis for the use of NLR in TBI for bleeding, neurosurgical management, and outcome determination. The goal of this study is to define the prognostic role of NLR in a TBI patient. We seek to demonstrate the efficacy of NLR as an objective, low-cost, and accurate measure of risk for secondary injury due to TBI.

## 2. Methods

### 2.1. Eligibility Criteria

This systematic review was reported following the Preferred Reporting Items for Systematic Reviews and Meta-Analyses (PRISMA) [22]. We included all studies involving: (1) report of the NLR for the study sample and (2) TBI-afflicted patients. Case series, cohort studies, non-randomized controlled trials, and randomized controlled trials were all eligible for inclusion. The exclusion of articles was based on the following criteria: (1) meta-analyses or systematic reviews, (2) describes pathologies other than TBI, (3) data was insufficiently disaggregated to extrapolate NLR for relevant cohorts.

### 2.2. Screening of Studies

A literature search of the PubMed, Scopus, and Web of Science (WOS) databases was conducted on 11 May 2022 to identify articles reporting neutrophil to lymphocyte (NLR measurements for patients with traumatic brain injuries. The same search strategy “((neutrophil to lymphocyte ratio) OR NLR) AND (intracerebral OR intracranial OR cerebral OR brain OR head) AND (TBI OR traumatic brain injury OR trauma)” was used in all three databases and was developed by one reviewer (AN). Study selection was performed independently by two reviewers (AN, TH) utilizing the Rayyan—Intelligent Systematic Reviews program [22]. Articles with potentially relevant titles and abstracts based on the inclusion criteria were included. Simultaneously, these articles were screened for correct interventions, study types, and outcomes to determine eligibility for full-text review. Data extraction was conducted on included full-text articles. When disagreements arose regarding study selection, a third reviewer (MD) acted as a mediator. Rayyan was used as a collaborative interface to record reasons for study exclusion following the independent screening.

### 2.3. Qualitative Analysis

The Newcastle-Ottawa scale (NOS) was used for risk of bias assessment [23]. The NOS criteria allowed for a maximum of four stars in the selection, two stars in comparability, and three stars in the outcome: the total range was 0–9. Case series were analyzed with NOS cohort guidelines without the application of comparability questions, making their effective range 0–7. Cohort and case-control studies were analyzed in full with a maximum score of 9. Two reviewers (TH, NG) conducted this assessment. The scoring is shown in Table 1.

### 2.4. Data Charting Process and Data Items

Data was collected independently by three reviewers (AN, TH, HL). Data items collected included: author, publication year, country of study, study type, number of patients, patient characteristics. This data was pooled for non-surgical vs surgical, bleeding vs no bleeding, and favorable vs unfavorable outcome cohorts. Individual studies reported bleeding upon imaging indicating intracranial bleeding including epidural hemorrhage, subdural hemorrhage, subarachnoid hemorrhage, or intracerebral bleeding including intraparenchymal hemorrhage. Unfavorable outcomes were defined by studies as Glasgow Outcome Scale (GOS) of 1–3 including death, while favorable outcomes were GOS 4–5. NLR was measured on the day of admission to the hospital or ED by included studies. If NLR values at various time points were provided, the value on the day of admission or day 1 was used. Presenting symptoms were also recorded when present. If any relevant clinical features were not reported in an article, it was assumed that this data was not present in that patient set. Weighted means were derived for data when applicable.

### 2.5. Statistical Analysis

Data analysis was conducted with RStudio programming software. Meta-analysis was utilized to express effect sizes with the standardized mean difference (SMD). A *p*-value < 0.05 was considered to be significant. Heterogeneity between studies was assessed with I^2^ derived from Cochran’s Q, due to the lower number of studies in the meta-analysis. If I^2^ > 50%, a random-effects model was chosen.

## 3. Results

**Figure 1:**
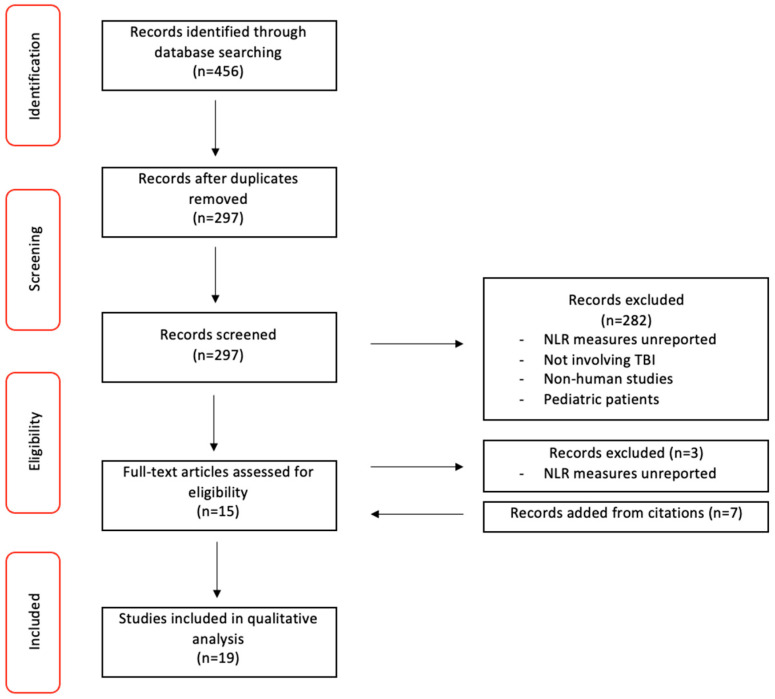
PRISMA Study Selection Flow Diagram.

We show the study selection process used in Figure 1. The initial literature search identified 456 articles for inclusion. Further screening yielded 159 duplicates across the three databases used (PubMed, Scopus, WOS), which were subsequently removed. Of the remaining 297 original studies, 281 were excluded as they did not satisfy inclusion criteria. The remaining 15 studies were included based on simple title and abstract screening using Rayyan. A citation scan of the 15 studies revealed 7 potential articles to be added. Full-text review then identified another 3 articles to be excluded due to their absence of a mean or median NLR report. The final 19 articles to be included for data extraction included 3 non-matched, case-control studies, 13 retrospective cohort studies, 1 prospective cohort study, 1 mixed (prospective and retrospective) cohort study, and 1 case series. (Table 2).

### 3.1. Qualitative Assessment

The studies were assessed with appropriate guidelines to characterize their quality based on several criteria (Table 1) [8,17,23,24,25,26,27,28,29,30,31,32,33,34,35,36,37,38,39]. NOS scores of 7–9 were deemed sufficient for the study as it fell in the region of “high quality” study according to the NOS guidelines, including case-control studies. For case series which had a maximum of 7, sufficient studies included those dissatisfying only 1 item per domain at maximum, with a total score of 5/7.

### 3.2. Demographics

In total, 7508 individuals were analyzed in the patient set. The mean age was 46.25 ± 9.77 years. Gender was reported for all patients, of which 73% were male (n = 5487) (Table 2).

### 3.3. Symptomatic Presentations

Symptomatic presentations were noted in nine of the included studies. Reported symptoms included headache, vomiting, neurological deficit, LOC, amnesia, psychomotor agitation, hypotension, pupillary abnormality (including unilateral and bilateral pupillary dilation and fixation), and seizures. Of the 4024 patients in studies that reported patient symptoms, 2.73% were reported to have a headache, 0.67% were reported to have vomiting, 11.46% were reported to have neurological deficit(s), 3.23% were reported to have LOC, 3.11% were reported to have amnesia, 0.35% were reported to have psychomotor agitation, 4.87% were reported to have hypotension, 16.30% were reported to have a pupillary abnormality, and 2.88% were reported to have a seizure. GCS at presentation was reported in 17 of the included studies, among which 15 were reported as a mean without stratification. Among these studies, average GCS at presentation was 10.51 ± 0.94 (Table 3).

### 3.4. Surgical Outcomes

**Figure 2:**
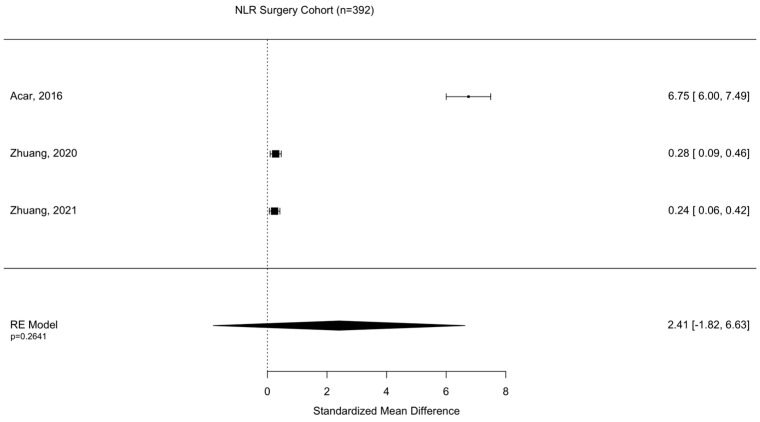
Assessment of NLR between surgical and non-surgical patient cohorts. Acar, 2016 [23]; Zhuang, 2020 [33]; Zhuang, 2021 [36].

**Figure 3:**
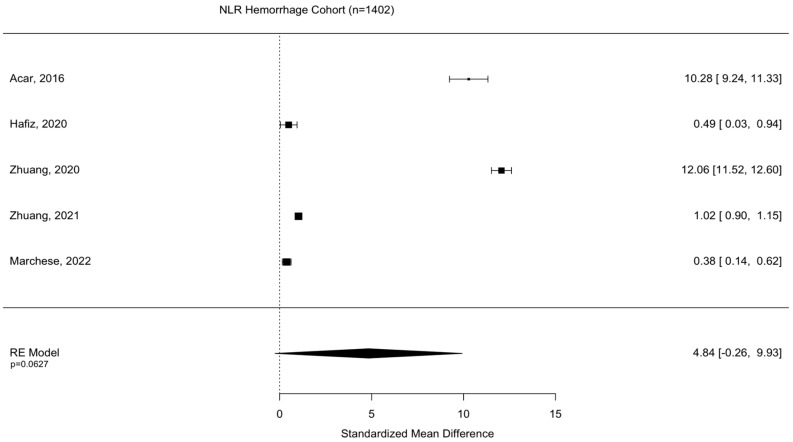
Assessment of NLR between bleeding and non-bleeding patient cohorts. Acar, 2016 [23]; Hafiz, 2020 [31]; Zhuang, 2020 [33]; Zhuang, 2021 [36]; Marchese, 2022 [38].

**Figure 4:**
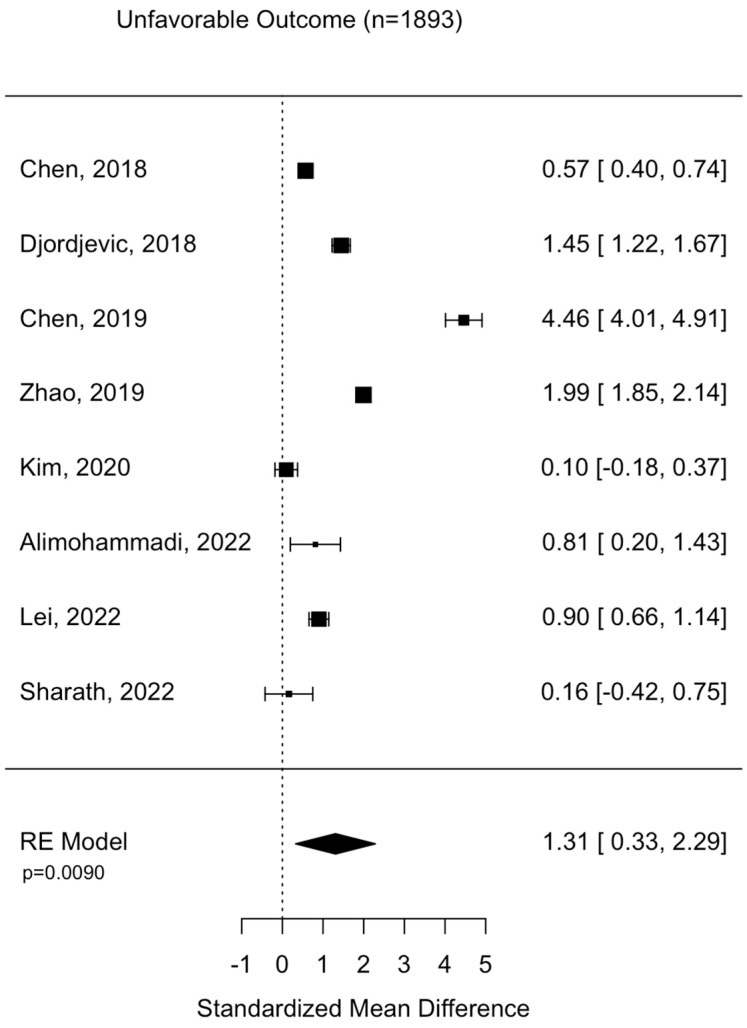
Assessment of NLR between favorable and non-favorable patient cohorts. Chen, 2018 [24]; Djordevic, 2018 [25]; Chen, 2019 [17]; Zhao, 2019 [27]; Kim, 2020 [29]; Alimohammadi, 2022 [37]; Lei, 2022 [39]; Sharath, 2022 [32].

Assessment of surgical intervention, favorability of outcome, and presence of intracranial bleeding among patients whose NLRs were reported were analyzed via formal meta-analysis. A total of 31.4% of patients were treated with surgery and there was no significant difference in the NLR between surgical vs. non-surgical cohorts (SMD 2.41 95% CI −1.82 to 6.63 *p* = 0.264) (Figure 2). A total of 52.4% of patients were observed with intracranial or intracerebral hemorrhage and there was no significant difference in the NLR between bleeding vs. non-bleeding cohorts (SMD 4.84 95% CI −0.26 to 9.93 *p* = 0.0627) (Figure 3). A total of 49.1% of patients experienced an unfavorable outcome and there was a significant increase in the NLR between favorable vs. unfavorable cohorts (SMD 1.31 95% CI 0.33 to 2.2 *p* = 0.0090) (Figure 4) (Table 4).

## 4. Discussion

Neutrophil-to-lymphocyte ratio is an emerging prognostic predictor of patient outcomes in various pathologies, among which primarily are cases of bacterial infection and inflammatory processes [40]. Its significance in TBI is not well understood due to uncertainty in the precise biological process that lymphocytes mediate in the brain following such injuries [20,40]. In cases of general tissue injury, neutrophils are among the first cellular responders, mediating destruction of pathological specimens and initiating an initial inflammatory response [41]. Within a week, the cellular majority shifts towards macrophages that have a role in cellular repair and fibrosis. The cytokines and biological alterations at the site of injury have been hypothesized to activate T-lymphocytes and further induce healing processes [42]. Given the different functions of neutrophils and lymphocytes, the prognostic function of the different compositions of both cellular types cannot be understated [40]. A lower NLR would be suggestive of greater lymphocyte counts that are associated with cellular repair. A higher NLR would suggest the contrary, a perpetual state of acute inflammation associated with high neutrophil counts [40]. The latter has been found to be correlated with poorer outcomes and is commonly found in higher-severity TBI cases [40]. The objective of our review was to examine the association between patient NLR measures obtained at initial presentation and admittance for isolated head trauma and secondary TBI outcome variables such as intracranial or intracerebral hemorrhaging including hematoma, surgical treatment requirement, and favorable vs unfavorable outcomes.

Neutrophils are generally thought to be the first responders to sites of tissue injury and mediate an innate immune response by way of phagocytosis and degranulation. Both processes actively recruit other leukocytes to action through cytokine and chemokine signaling [43]. At this point, cells such as macrophages/monocytes work to destroy pathogens, phagocytose, and clear debris [44]. The immune system then progresses towards an adaptive response with the activation of B and T (CD 4, CD8) lymphocytes with the help of antigen-presenting cells [45]. This simplified immune response timeline normally ends with the clearance of these cells along with dampened recruitment through inflammation suppression. The overall process of acute inflammation suppression and tissue healing after infection, infiltration, etc. is a rather complicated interplay between a variety of mediators, in which many leukocytes including macrophages and lymphocytes signal both pro-inflammatory and anti-inflammatory cytokines [46]. Two well-studied opposing profiles include the M1/M2 macrophage and Th1/Th2 helper T cell balance. The former cell types are responsible for inducing inflammation and cytotoxicity, while the latter cell types generally promote tissue healing and reduced inflammation [47,48]. Additionally, regulatory T cells/suppressor T cells also contribute to the downregulation of immune responses. Their role is particularly integral to autoimmune disorders [49]. Given that multiple cell types are involved in both propagating and terminating acute immune responses, we cannot simply use NLR as a substitute measure for quantifying acute inflammation. However, that does not mean NLR does not provide any insight into the progression of an acute immune response.

A large neutrophil count might indicate inflammation levels are elevated from baseline based on two concepts: 1. an acute immune response is likely in its initial stages and 2. neutrophils are generally unidirectional in promoting an inflammatory response [50]. Although it is not known how well NLR is directly correlated with inflammation, our results did show a significant difference in NLRs between patients who experienced favorable outcomes (GOS of 4–5) post-TBI and those who experienced unfavorable outcomes (GOS of 1–3). With regards to evaluations of unfavorable versus favorable outcomes for TBI patients, the Glasgow Outcome Scale (GOS) has not only been one of the oldest standard measures by which clinicians have assessed acute closed head injuries, but also one of the most popular ones as well [51]. The GOS is an ordinal scale for evaluating TBI patients measured at discharge [37]. The measure delineates patient outcomes into categories of Death (1), Persistent Vegetative State (2), Severe Disability (3), Moderate Disability (4) and Good Recovery (5) [37]. These values can subsequently be dichotomized into unfavorable outcomes (categories 1–3) and favorable outcomes (4–5) [37]. Its prognostic application, marked inter-rater reliability, and validity have been explored and refined over the last 40 years, and its ease of use and utility have allowed it to serve for numerous clinical guidelines for TBI cases over alternative scales, such as both the disability rating scale (DRS) and the Barthel Activities of Daily Living index (ADL) [52]. Additionally, studies have found it to outrank DRS measures in correlations to self-reported measures of depression, mental well-being and neurobehavioral and functioning outcomes in patients suffering from TBIs [53].

While the precise mechanism remains to be elucidated, our study aims to see if NLR can reliably be used as a marker of TBI severity specifically. Of note, the pooled results of our study showed a significantly higher NLR among TBI patients with poor clinical outcomes. As previously stated, our included studies defined unfavorable outcomes as a GOS score of 1–3 including death, while favorable outcomes were considered a GOS score of 4–5 upon discharge. Both the Glasgow Outcome Scale (GOS) and the Glasgow Coma Scale (GCS) is the current Gold Standard prognostic test for outcomes in patients with TBI.55 Similar to GOS, GCS is a widely accepted and commonly reported outcome measure for head trauma, with only a few modifications over the years. Convenience, accessibility, and overall effectiveness are true of both these measures [51].

Correlation between NLR and GCS has been found with both reliably assessing outcomes for patients with mild TBI [46]. However, previous studies have shown that NLR has a similar if not more objective predictive value given that its measurements do not rely on subjective measurements of patient well-being, decreasing the possibility of human error [54,55]. NLR measurements are also independent of the patient’s ventilation status, state of consciousness, and other factors that might affect how accurately a measurement can be taken with GCS or GOS. Should NLR be found to have the same prognostic power as GCS or GOS in TBI patients, this would provide health professionals with another objective lab test option predictive of outcomes specific to TBI severity. As such, NLR could have immense clinical utility in the early medical management and subsequent treatment course of patients with TBI. In a clinical setting and if more widely studied, it could be argued that the benefits of correlation between NLR and outcome measures outweigh the lack of a known mechanism. There are, however, well-studied blood-based biomarkers specific to neuroinflammation [56]. Unfortunately, these measures are not readily available from routine tests including complete blood count (CBC) with differential [57]. Further studies are needed to shed more understanding on how NLR relates to TBI outcomes. In a shorter time frame, it might be possible to implement NLR in conjunction with other measures suggestive of inflammation, infection, injuries such as fever, C-reactive protein (CRP), erythrocyte sedimentation rate (ESR), or plasma viscosity (PV) in order to circumvent the time and costs of longer and more costly tests/imaging.

Initial bleeding in the brain and prolonged vessel leakage is critical TBI outcome measures because they compromise blood-brain barrier (BBB) integrity. The BBB refers to the collective endothelial cell layer lining the capillaries of the central nervous system (CNS) [58]. Since the site of exchange between the brain and perfusing arteries occurs at the level of the capillaries, the structure and function of the endothelial cells largely determine which molecules and ions are free to enter and exit the CNS. The tight junctions that connect these cells ensure an extremely low rate of transfer between the brain and peripheral vascular system with the exception of highly specific transporters [54]. Interestingly, leukocyte adhesion molecules are expressed at very low levels by these endothelial cells, suggesting that the prevention of immune cell entry into the CNS is a strong indicator of the proper functioning and maintenance of the BBB [55]. On the other hand, injuries to blood vessels and associated cell linings result in an elevation of leukocyte extravasation through endothelial cells and into brain tissue [59]. BBB breakdown also allows for the passage of many damaging infiltrates in addition to peripheral immune cells including reactive oxygen species (ROS), increased microglia and astrocytes, and water [60]. Rapid accumulation of fluid around the brain can lead to sustained cerebral edema if not naturally restored by normal BBB filtration or rescued through surgical intervention [61]. Leukocyte-cytokine signaling and subsequent inflammation contribute to impairment of the cellular repair vital to restoring BBB integrity [59]. Without this normal filtration process, uncontrolled cerebral edema and intracranial pressure (ICP) in conjunction with elevated secondary inflammation allow intracranial hemorrhaging and hematomas to continue and expand respectively [62]. In addition to the opportunistic infiltration afforded by trauma to the central nervous system (CNS), neutrophils also contribute to neural damage by way of neutrophil extracellular traps (NETs), which are structures released by neutrophils aimed to trap then neutralize or eliminate pathogens [63]. As a byproduct, NETs generate an overabundance of harmful cytotoxic proteins, further interfering with cellular repair [64]. NET dysregulation has been implicated in pathologies ranging from autoimmune disorders such as psoriasis to cancers, trauma, and neurodegenerative diseases.

Our analysis of 1402 patients for which the presence or absence of intracranial hemorrhaging (epidural hemorrhage, subdural hemorrhage, subarachnoid hemorrhage) including hematoma or intracerebral bleeding including intraparenchymal hemorrhage was confirmed with head CT showed no significant difference in NLR measures at the first day of admission or hospital course. This evidence would suggest initial NLRs do not strongly correlate with post-TBI hemorrhaging, however, this discrepancy might be attributable to the nuanced physiological changes reflected by the development of traumatic brain bleeds. When severe enough, TBI can produce enough irritation, damage, and edema to result in blood vessel damage and sudden onset intracranial bleeding [65,66]. As discussed earlier, NLR might not independently predict inflammation, but it does provide temporal orientation as to the current immune response timeline. The NRLs used in our data analysis were consistently collected early on in each patient’s course of stay, but that time point relative to when the TBI incident occurred was not consistently controlled for among our included studies. An NLR obtained directly after the TBI could possibly correlate significantly more than otherwise. Although NLR is inherently time-constrained, it still might prove useful in relation to brain bleed recovery. The previously outlined mechanism detailed how increased secondary inflammation, suggested by high neutrophil count and low lymphocyte count (more innate/less adaptive immune response), impairs BBB cellular repair and allows for further bleeding development. It is possible NLR correlates significantly more with brain bleed progression than the presence or absence of hemorrhaging itself. This might explain why there was not a statistically significant difference in NLRs between patients who experienced intracranial bleeding and those who did not, yet there was a statistically significant difference in NLRs between patients who experienced favorable outcomes and those who did not. Additionally, our meta-analysis included some studies that excluded cases of moderate to severe TBI, thereby limiting the full range of intracranial hemorrhaging-associated NLRs available for analysis. Mild TBI cases may result in delayed/subacute or chronic microhemorrhage/hematoma if at all [23,67]. It would make sense that less prominent disturbances would be less sensitive to shifts in NLR. Again, due to the inherent limitations and conditions of using NLR alone, it would be more clinically effective to corroborate with other tests such as a D-dimer test or a mean platelet volume (MPV) blood test. Both a large D-dimer and MPV value indicate possible clot formation including deep vein thrombosis (DVT), pulmonary embolism (PE), or more importantly traumatic ischemic stroke [68,69]. These pathologies can be ruled out with a D-dimer or MPV value within normal limits, however, they would not be able to definitively indicate the presence of traumatic hemorrhagic stroke as a secondary outcome to TBI [70]. Interestingly, Acar et al. found a significant difference between high troponin levels in TBI patients with and without brain pathology, as there may be cardiovascular compensation in severe cases of TBI [23,71].

Unsurprisingly our results showed there was no significant difference in NLR values between patients who did and patients who did not undergo surgical treatment for TBI-related complications. This evidence suggests that patients who are being examined for acute head trauma are either surgically treated or only medically treated independent of their NLRs at the initial encounter. One probable explanation for this inconsistency could be the subjective approval required prior to any surgery. Even if recommended, the decision to proceed with surgery ultimately falls upon the patient. Furthermore, the management of traumatic intracerebral hemorrhage and acute subdural hematoma in TBI cases is not equipped with clear protocols in place. Craniotomy, craniectomy, and trephination are the most commonly elected procedures for effective hemorrhage and/or hematoma evacuation in TBI patients [72]. There is still debate as to whether early surgical intervention to potentially prevent further blood extravasation, neurotoxicity, and secondary tissue damage is preferable to conservative medical treatment and observation for deterioration [73]. Although surgery has been shown to prevent further brain damage, some evidence has pointed to benefits in mainly moderate cases of injury. Mild cases might have the flexibility for observation, whereas severe cases present a significant risk for intraoperative and postoperative complications [74]. These surgeries are especially indicated for when accompanied by high intracranial pressure (ICP) changes. Depending on resources, consistent ICP monitoring is not always readily available at every institution [75]. Conversely, NLR is readily available, and if additional studies showed strong evidence for an association with TBI surgical indication, surgical management of these cases would be much more streamlined.

Surgical intervention and hemorrhaging/hematoma are both parameters that depend on each other to an extent, namely the former is a response to the latter. Therefore, some of the same study limitations apply to using NLR as a correlating measure for both aspects of TBI, specifically inconsistent TBI severity inclusion. Not all moderate to severe TBI cases were included across all our studies. This limits our surgical sample size and external validity but also presents as a potential confound. As presently studied, NLR may or may not be a strong marker for inflammation, but there is a known neutrophil role within the inflammatory recruitment response. Lower threshold NLRs alone from higher severity outcomes such as bleeding and surgery may not be as compatible as with minor outcomes. However, as stated there is a subjective and objective component to proceeding with surgical options when faced with a traumatic intracranial bleed, and these obstacles may also be linked to discrepancies in NLR correlation with surgical intervention.

Due to the aforementioned benefits, the prognostic value of NLR in relation to TBI warrants further investigation. However, it is important to note the limitations of our study for future research efforts. First, the NLRs’s in our included studies had varying timepoints in which the values were recorded. While the NLR values collected and used for this study were on the day of admission or the first day of the hospital course, patients’ clinical progression following TBI occurrence could not be controlled for. Therefore, it would be valuable in future analyses to contextualize these differences, perhaps including only those patients presenting within the same day of the trauma incident. Due to the time-sensitive clinical progression of patients with TBI, it is important for future studies to have consistent times NLRs are recorded in order to standardize values for prognostic outcomes. In a similar vein, the pooled participant sample from included studies was heterogeneous in terms of patient age and TBI severity. Similar research efforts have shown age to be a confounding variable when analyzing NLR’s utility in risk assessment [76]. In particular, a patient’s age was correlated with baseline NLR values thus affecting the prognostic value of post-NLR values used. Considering this impact, future research efforts to examine this relationship would be beneficial for data standardization and clinical application.

Furthermore, studies applied variant criteria in patient inclusion—while the majority excluded cases involving immunosuppressive conditions, major heart/systemic illnesses, prior brain trauma, and strokes, a few did not. Comorbidities such as hypertension and diabetes were not controlled for in studies and therefore, this should be acknowledged when interpreting the results of both the individual studies and the current analysis. Pre-existing immunosuppressive conditions present a particular challenge to correlating NLRs with secondary TBI outcomes relating to inflammatory-based damage and repair interference. Chronic inflammation in an aging population, known as inflammaging, has been linked to a variety of chronic conditions including neurodegenerative diseases [77]. If a patient’s neutrophils are already elevated at baseline, then NLR loses its temporal resolution. It becomes more difficult to determine if a patient is presenting with an earlier or later acute immune response as a direct response to TBI because of background inflammation and the accompanying physiological standards for that one patient. The progression from innate to adaptive immune response, including lymphocytes, within the context of chronic inflammation, is as well understood as a short-term acute response. Transitions such as M1/M2 macrophage and Th1/Th2 do not necessarily proceed in the same manner, and immune cell roles are less well understood in these cases [78]. Although in these cases NLR is less useful as an inflammatory marker, it might still provide temporal insight when used as a comparative marker to other NLRs obtained with the same hospital course. Assuming there are no interactions between chronic and acute inflammation, the difference between two NLRs short-term would theoretically cancel out background levels of inflammation, neutrophil and lymphocyte levels, etc. Admittedly, this is an oversimplified solution to this issue of pre-existing comorbidities. Practically speaking, it would make more sense to corroborate NLR with other measures to compensate where NLR fails to succeed. These limitations addressed within the paper have also been reflected within measures of the GOS. Measurements via GOS evaluations have been noted to lack comprehensive consideration of both patient heterogeneity and underlying comorbidities [79,80]. As such, efforts have been made to expand the dichotomy of unfavorable to favorable outcomes from a fixed scale to a sliding scale that accounts for previous patient histories, but the implementation of such analyses into evaluations of patient outcomes is ongoing, and may be the subject of future evaluations of TBI outcomes [80,81]. Additionally, regarding symptomatic presentation, the majority of included studies did not report some or all of the symptoms that were recorded in our review, or reported this data in a format that could not be accurately translated to a binary yes-or-no format (e.g., reporting LOC as a duration of <30 min., 30 min. to 24 h., and >24 h. without specifying the number of patients in the <30 min. category that did not experience LOC altogether). As a result, the proportion of symptoms observed in our review is likely an underestimation of the true proportion of patients with these symptoms, particularly skewed toward symptoms that were more commonly discussed.

## Figures and Tables

**Table 1:** Newcastle-Ottawa Scale Qualitative Analysis.

		Selection				Comparability	Outcome			
Cohort/Case Series										
Author and Year	Study Type	Representativeness of the exposed cohort	Selection of the non-exposed cohort	Ascertainment of exposure	Demonstration that outcome of interest was not present at the start of the study	Comparability of cohorts on the basis of the design or analysis	Assessment of outcome	Was follow-up long enough for outcomes to occur	Adequacy of follow-up of cohorts	Total
Chen, 2018 [24]	RC	1	1	1	1	0	1	1	1	7
Djordevic, 2018 [25]	PC	1	1	1	1	0	1	1	1	7
Chen, 2019 [17]	RC	1	1	1	1	0	1	1	1	7
Ge, 2022 [26]	RC	1	1	1	1	0	1	0	0	5
Zhao, 2019 [27]	RC	1	1	1	1	0	1	1	1	7
Alexiou, 2020 [28]	RC	1	1	1	1	0	1	0	1	6
Kim, 2020 [29]	RC	1	1	1	1	0	1	1	1	7
Kusuma, 2020 [30]	CS	1	N/A	1	1	NA	1	0	1	5
Hafiz, 2020 [31]	RC	1	1	1	1	0	1	1	0	6
Sharath, 2022 [32]	PC	1	1	1	1	0	1	0	0	5
Zhuang, 2020 [33]	RC, PC	1	1	1	1	0	1	0	1	6
Bail, 2021 [34]	RC	1	1	1	1	0	1	0	1	6
Chen, 2021 [35]	RC	1	1	1	1	0	1	0	1	6
Zhuang, 2021 [36]	RC	1	1	1	1	0	1	0	1	6
Alimohammadi, 2022 [37]	RC	1	1	1	1	0	1	0	1	6
Defort, 2022 [8]	RC	1	1	1	1	0	1	0	1	6
Case-Control										
		Selection				Comparability	Outcome			
Author and Year	Study Type	Is the case definition adequate	Representativeness of the cases	Selection of Controls	Definition of Controls	Comparability of cases and controls on the basis of the design or analysis	Ascertainment of exposure	Same method of ascertainment for cases and controls	Non-Response rate	Total
Acar, 2016 [23]	CC	1	1	1	1	0	1	1	1	7
Marchese, 2022 [38]	CC	1	1	1	1	0	1	1	1	7
Lei, 2022 [39]	CC	1	1	1	1	0	1	1	1	7

**Table 2:** Demographic variables of studies.

Author, Year	Study Design	Country	Age (Years)	Age SD	Male	Female
Acar, 2016 [23]	Case-control	Turkey	NA	NA	151	49
Chen, 2018 [24]	Retrospective Cohort	China	45.4	14.85	557	131
Djordevic, 2018 [25]	Prospective Cohort	Serbia	53.67	18.26	236	156
Chen, 2019 [17]	Retrospective Cohort	China	56	NA	256	60
Zhao, 2019 [27]	Retrospective Cohort	China	47.03	16.88	982	309
Alexiou, 2020 [28]	Case-control	Greece	61.6	19.9	85	45
Kim, 2020 [29]	Retrospective Cohort	South Korea	56.77	NA	155	45
Kusuma, 2020 [30]	Cross-sectional	Indonesia	38.89	15.27	66	19
Hafiz, 2021 [29]	Retrospective Cohort	Indonesia	32.34	21.67	64	64
Zhuang, 2020 [30]	Mixed Retrospective and Prospective Cohort	China	48.3	NA	764	239
Bail, 2021 [34]	Retrospective Cohort	France	54	NA	88	27
Chen, 2021 [35]	Retrospective Cohort	China	44.5	NA	113	19
Zhuang, 2021 [36]	Retrospective Cohort	China	48.63	17.92	815	262
Alimohammadi, 2022 [37]	Retrospective Cohort	Iran	7.37	3.11	197	197
Defort, 2022 [8]	Retrospective Cohort	Poland	46.37	18.7	79	16
Ge, 2022 [26]	Retrospective Cohort	China	53.27	16.27	663	329
Lei, 2022 [39]	Case-control	China	51.1	15.2	33	12
Marchese, 2022 [38]	Case-control	Italy	11.54	4.79	150	69
Sharath, 2022 [32]	Prospective Cohort	India	63.37	NA	31	29
Total or Mean			46.25	9.77	5487	2021

**Table 3:** Symptomatic characterization.

Presenting Symptom	Percentage
Headache	2.73%
Vomiting	0.67%
Neurological Deficit	11.46%
LOC	3.23%
Amnesia	3.11%
Psychomotor Agitation	0.35%
Hypotension	4.87%
Pupillary Abnormality	16.30%
Seizure	2.88%

**Table 4:** Analysis of outcomes.

	Surgery	Bleed	Outcome (Unfavorable)
**Total/Average**	31.4%	52.4%	49.1%
**Overall effect**	SMD 2.41 95% CI −1.82 to 6.63 *p* = 0.2641	SMD 4.84 95% CI −0.26 to 9.93 *p* = 0.0627	SMD 1.31 95% CI 0.33 to 2.29 *p* = 0.0090

## Data Availability

The data presented in this study are available in cited references.

## Abbreviations

TBI = traumatic brain injury, LOC = loss of consciousness, GCS = Glasgow Coma Scale, NLR = neutrophil-to-lymphocyte ratio, GOS = Glasgow Outcome Scale, BBB = blood brain barrier, ROS = reactive oxygen species, NETs = neutrophil extracellular traps, MPV = mean platelet volume.
